# Inhibition of human carboxylesterases by ginsenosides: structure–activity relationships and inhibitory mechanism

**DOI:** 10.1186/s13020-019-0279-0

**Published:** 2019-12-16

**Authors:** Zhao-Hui Sun, Jing Chen, Yun-Qing Song, Tong-Yi Dou, Li-Wei Zou, Da-Cheng Hao, Hai-Bin Liu, Guang-Bo Ge, Ling Yang

**Affiliations:** 1 Institute of Interdisciplinary Integrative Medicine Research, Shanghai University of Traditional Chinese Medicine, Shanghai, 201203 China; 2 School of Life Science and Medicine, Dalian University of Technology, Panjin, 124221 China; 3 School of Environment and Chemical Engineering, Dalian Jiaotong University, Dalian, 116028 China; 4 National Engineering Research Center for Gelatin-based Traditional Chinese Medicine, Dong-E-E-Jiao Co. Ltd., Liaocheng, 252201 China

**Keywords:** Ginsenosides, Human carboxylesterases (hCES), Structure–inhibition relationships, Selectivity, Inhibitory mechanism

## Abstract

**Background:**

Human carboxylesterases (hCES) are key serine hydrolases responsible for the hydrolysis of a wide range of endogenous and xenobiotic esters. Although it has been reported that some ginsenosides can modulate the activities of various enzymes, the inhibitory effects of ginsenosides on hCES have not been well-investigated.

**Methods:**

In this study, more than 20 ginsenosides were collected and their inhibitory effects on hCES1A and hCES2A were assayed using the highly specific fluorescent probe substrates for each isoenzyme. Molecular docking simulations were also performed to investigate the interactions between ginsenosides and hCES.

**Results:**

Among all tested ginsenosides, Dammarenediol II (DM) and 20S-*O*-β-(d-glucosyl)-dammarenediol II (DMG) displayed potent inhibition against both hCES1A and hCES2A, while protopanaxadiol (PPD) and protopanaxatriol (PPT) exhibited strong inhibition on hCES2A and high selectivity over hCES1A. Introduction of *O*-glycosyl groups at the core skeleton decreased hCES inhibition activity, while the hydroxyl groups at different sites might also effect hCES inhibition. Inhibition kinetic analyses demonstrated that DM and DMG functioned as competitive inhibitors against hCES1A-mediated d-luciferin methyl ester (DME) hydrolysis. In contrast, DM, DMG, PPD and PPT inhibit hCES2A-mediated fluorescein diacetate (FD) hydrolysis via a mixed manner.

**Conclusion:**

The structure–inhibition relationships of ginsenosides as hCES inhibitors was investigated for the first time. Our results revealed that DM and DMG were potent inhibitors against both hCES1A and hCES2A, while PPD and PPT were selective and strong inhibitors against hCES2A.

## Background

**Fig. 1:**
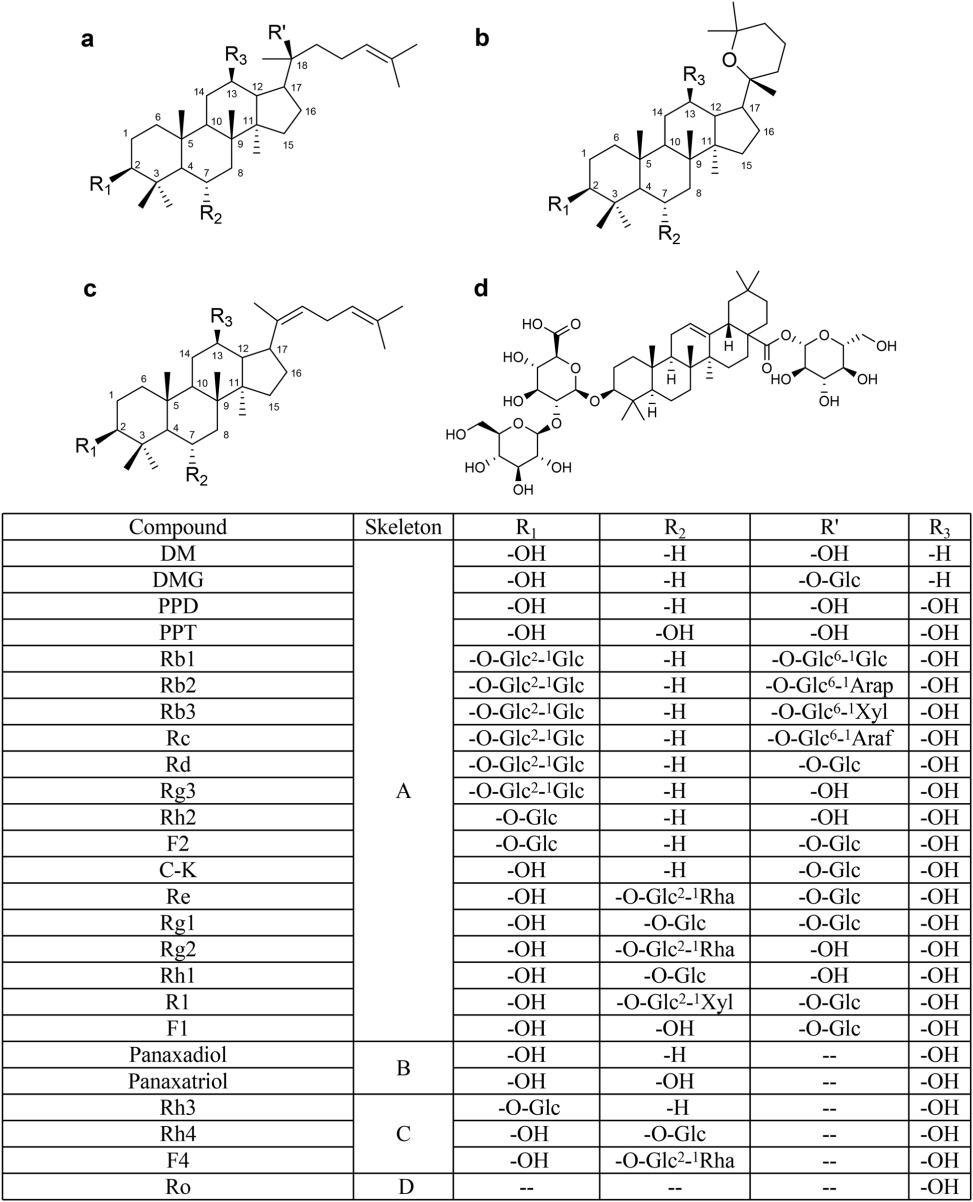
The chemical structures of 25 ginsenosides from ginseng. *Glc* β-d-glucopyranosyl, *Arap* α-l-arabinopyranosyl, *Araf* α-d-arabinofuranosyl, *Rha* α-l-rhamnopyranosyl, *Xyl* β-d-xylopyranosyl. Numerical superscripts indicate the carbon in the sugar ring that links the two carbohydrates

Ginseng, the root or rhizome of *Panax ginseng* Meyer, one of the most popular edible herbs used in both eastern and western countries, has been found with many beneficial effects for human health. Modern pharmacological and pharmacodynamic researches have demonstrated that both ginseng extract and its major constituents (ginsenosides) can enhance memory, improve immunity, improve cardiovascular functions, delay aging and anti-tumor, etc. [1–6]. Over the past two decades, the pharmacological activities of ginseng products and its major constituents have been extensively investigated and reported [7–10]. Ginsenosides, as the major bioactive constituents in ginseng, have been proven to have salient effects on immunomodulation [5], anti-tumor [11–14] and anti-inflammatory activities [15, 16]. Until now, more than 20 ginsenosides have been identified from *Panax ginseng*, and most of them are 20(S)-protopanaxadiol (PPD)-type and 20(S)-protopanaxatriol (PPT)-type ginsenosides [17] (Fig. 1).

In many Asia countries, ginseng has been widely used as a key material for preparing dietary supplements, herbal medicines and cosmetics [18, 19]. Currently, a wide variety of ginseng products have been marketed (such as Spirit of Ginseng, Ginseng Bolus for Tonifying Spleen) in Asia countries and its health-promoting effects have been well-accepted [18, 19]. Notably, many olds or patients take ginseng products daily, owing to they believe that this herb is very safe and it can regulate or balance most of the systems in the human body. Although ginseng products and most of ginsenosides have been found with excellent safety profiles, recent reports have shown that ginsenosides and its metabolites can modulate the treatment outcomes of some therapeutic drugs that can inhibit some key human drug metabolizing enzymes, including UDP-glucuronosyltransferase 2B7 (UGT2B7) and cytochrome P450 3A (CYP3A4) [20, 21]. In view of the wide applications of ginseng products and the combined use of ginseng products and clinical drugs, it is necessary to systematically examine the interactions of ginsenosides with human drug metabolizing enzymes. Our previous studies have reported that the intestinal bacterial metabolites of ginsenosides (such as C-K) can strongly inhibit CYP3A4 and UGT2B7, which may affect the metabolism of the drugs mainly metabolized by these enzymes [20, 21]. However, the interactions of natural ginsenosides or their bacterial metabolites with other key drug metabolizing enzymes (including the esterases) have not been well-investigated.

Mammalian carboxylesterases (CES) are key hydrolases in most organs, which catalyze the hydrolysis of various esters (including endogenous and xenobiotic ones) into the corresponding alcohols and carboxylic acids [22–24]. In the human body, human carboxylesterases 1A (hCES1A) and human carboxylesterases 2A (hCES2A) are two predominant isoenzymes that have been extensively investigated over the past decade [25, 26]. The substrate preferences of these two isoenzymes have been reported, hCES1A prefers to hydrolyze the ester substrates with small alcohol groups and bulky acyl groups, such as oseltamivir, imidapril, clopidogrel [27, 28]. By contrast, hCES2A like to hydrolyze the esters with relatively large alcohol groups and small acyl groups, such as irinotecan and flutamide [29, 30]. Several hCES2A substrates, such as the anti-cancer agent irinotecan, could induce severe intestinal toxicity that directly caused by the excessive production of SN-38 (the hydrolytic metabolite of irinotecan) in intestinal tract, while intestinal hCES2A is a key target to modulate the intestinal toxicity of this agent [31, 32]. Treatment with strong hCES2A inhibitors may partially block the production of SN-38 in intestinal tract, and then ameliorate the severity of diarrhea and improve the patient’s quality of life [33–37]. In addition, hCES also have pivotal effects on hydrolytic metabolism of some key endogenous esters, including triglycerides and cholesterol esters [38]. Thus, inhibition or dysfunction of hCES may strongly affect the treatment effects of hCES-substrate drugs, as well as the endogenous metabolism.

Over the past decade, ginseng-related products have been widely used in combination with a variety of therapeutic drugs to treat various types of cancer in clinical settings. In addition to enhancing efficacy and reducing drug toxicity, the accumulating evidence has indicated that ginseng products may bring benefits to the cancer patients, including increasing immunity and tolerance of cancer patient [13, 39]. Notably, it has been reported that Hange-shashin-to (a ginseng-containing Chinese herbal formula) could significantly relieve hCES2A-mediated diarrhea induced by irinotecan [40]. These findings inspired to investigate the modulatory effects of on the catalytic activities of hCES. To this end, more than 20 ginsenosides were collected and their inhibitory effects on both hCES1A and hCES2A were assayed. Meanwhile, the structure–inhibition relationships of ginsenosides as hCES inhibitors were also explored in this study. Furthermore, a set of molecular docking simulations and inhibition kinetic assays were performed to explore the inhibitory mechanism of some potent ginsenoside-type hCES inhibitors. These findings provide solid data to deeply understand the inhibition of hCES by ginsenosides, which are very helpful for the pharmacists to reasonable use ginseng-related products for alleviating CES-associated drug toxicity or avoiding the occurrence of hCES-mediated herb–drug interactions in clinical settings.

## Materials and methods

### Chemicals and reagents

Twenty-five ginsenosides were purchased from Chengdu Pufei Biotech Co., Ltd. (Chengdu, China) and used in this study. Luciferin Detection Reagent (LDR) was obtained from Promega Biotech (Madison, USA). Fluorescein diacetate (FD) was from TCI (Tokyo, Japan), *N*-(2-butyl-1,3-dioxo-2,3-dihydro-1*H*-phenalen-6-yl)-2-chloroacetamide (NCEN) and d-luciferin methyl ester (DME) was synthesized by us and the synthetic schemes have been reported previously [41, 42]. FD and DME were used as the highly specific probe substrates for hCES2A and hCES1A, respectively. The purity of all compounds tested in this study reaches more than 98% by LC-UV. The pooled human liver microsomes (HLMs, from 50 donors, lot no. X008067) obtained from Bioreclamation IVT (Baltimore, MD, USA), were used as the enzyme source for hCES inhibition assays. HepG2 cells were purchased from the American Type Culture Collection (Teddington, Middlesex, UK). Chromatographic grade DMSO (Tedia, USA) was used to prepare the stock solutions of all compounds. 0.1 M Phosphate buffer of pH 6.5 and pH 7.4 was used for hCES1A and hCES2A, respectively, which was configured by Millipore water (Millipore, Bedford, USA). All configuration solutions were stored at 4 °C until use.

### hCES1A inhibition assays

DME hydrolysis was used as the probe reaction to assay the inhibitory effects of 25 ginsenosides against hCES1A, oleanic acid was used as a positive control of inhibition assays [43]. The incubation system contains 91 μL PBS (0.1 M pH 6.5), 5 μL HLM (1 μg/mL, final concentration) and 2 μL corresponding inhibitor. After pre-incubating the reaction mixture for 3 min at 37 °C, the reaction was started by adding 2 μL DME (3 μM, final concentration) with a total volume of 100 μL, in which the final content of DMSO was limited to 2% (v/v). After incubating for 10 min at 37 °C in a shaking bath, LDR (50 μL, equal volume of the incubation mixture) was added to terminate the reaction. The luminescence signals of d-luciferin (the hydrolytic metabolite of DME) was measured (excitation wavelength 580 nm) by a multi-mode microplate reader (SpectraMax^®^ iD3, Molecular Devices, Austria). The calculation of residual activities of different inhibitors against hCES1A was performed as described previously [44].

### hCES2A inhibition assays

To determine the inhibitory effects of ginsenosides against hCES2A, FD was used as the probe substrate, while loperamide (LPA) was used as a positive control of hCES2A inhibition assays. The incubation system contains 2 μL HLM (1 μg/mL, final concentration), 194 μL PBS (0.1 M pH 7.4) and 2 μL corresponding inhibitor. After pre-incubating the incubation mixture for 3 min at 37 °C, the reaction was started by adding FD (2 μM, final concentration) with a total volume of 200 μL, in which the final content of DMSO was limited to 2% (v/v). The hydrolytic metabolite of FD was quantified (excitation wavelength 480 nm, emission wavelength 525 nm, the PMT gain value 500 V and integration time 10 ms) by a SpectraMax^®^ iD3 multi-mode microplate reader. The kinetic parameters were set at 30 reads, with an interval of 30 s (30 min) and shake during 5 s before the first read at 37 °C. The calculation method for residual activities of different inhibitors against hCES2A have been reported previously [37].

### Inhibition kinetic analyses

According to the inhibition kinetics of both hCES1A and hCES2A, which were investigated by using varying concentrations of probe substrates in the presence of different concentrations of ginseng compounds (inhibitors), the inhibition constant (*K*_*i*_) and inhibition kinetic types of ginsenosides were determined. The experimental details used to determine the inhibition constants have been reported previously [37, 45].

### Inhibition of hCES2A by PPT in living cells

To further verify whether the newly identified ginsenoside-type CES inhibitors could also inhibit intracellular hCES, the inhibition potentials of PPT (a highly specific and potent ginsenoside-type hCES2A inhibitor) on hCES2A were investigated in living cells. The detailed processes for hCES2A inhibition assays in HepG2 cells have been depicted previously [45].

### Molecular docking simulations

Docking simulations were executed to simulate the interactions between ginseng constituents and hCES by using Discovery Studio (BIOVIA Discovery Studio 2016, Dassault Systèmes, San Diego, USA). The detailed processes for docking simulation were depicted previously [37, 43].

### Statistical analysis

All inhibition assays were performed in triplicate, nonlinear regression was used to determine the IC_50_ and *K*_*i*_ values with GraphPad Prism 7.0 software (GraphPad Software, Inc., La Jolla, USA).

## Results

### Inhibitory potentials of ginsenosides on hCES

**Fig. 2:**
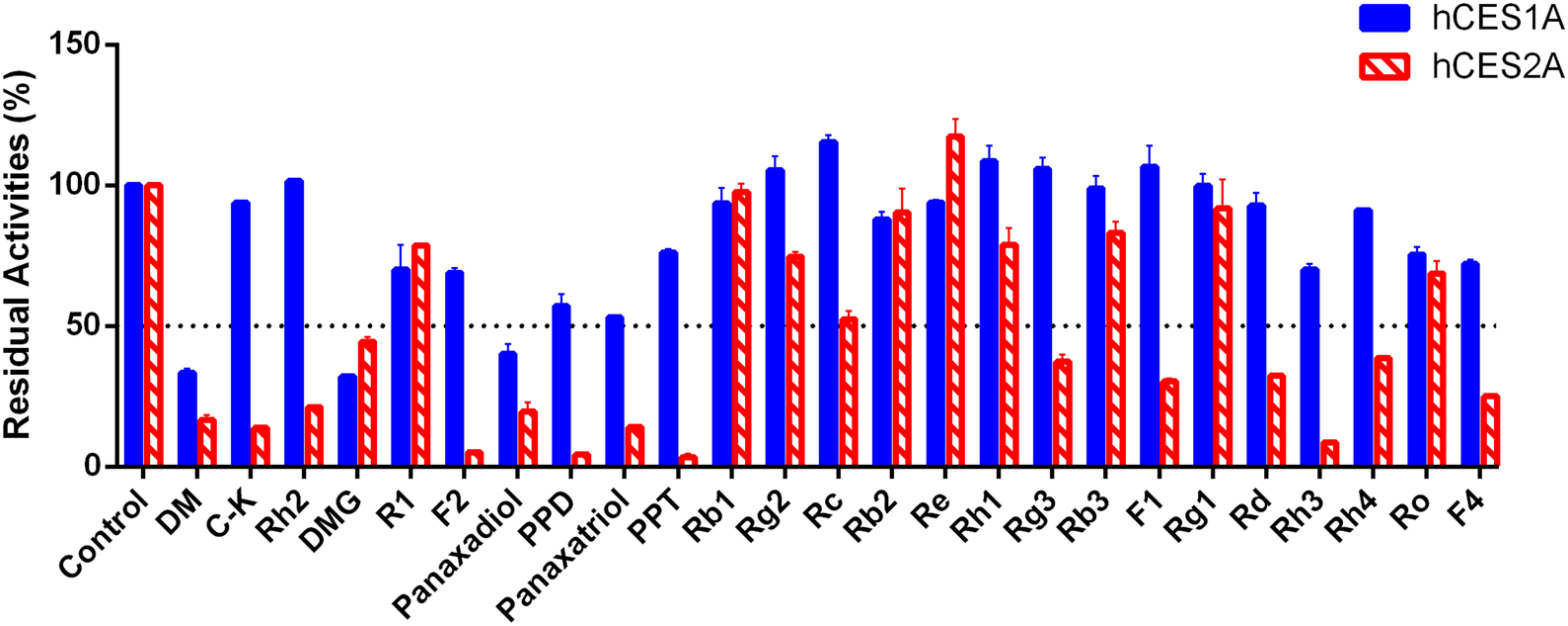
The residual activities of hCES1A and hCES2A upon addition of each ginsenoside. The final concentration of each ginsenoside was 100 μM. The vertical bar represents mean ± SD

First of all, the inhibitory potentials of 25 ginsenosides against hCES1A and hCES2A were preliminarily screened. As shown in Fig. 2, most constituents with the *O*-glycosyl groups on the core skeleton (e.g., ginsenosides Rb1, Rb2, Rb3, Rg1, Rg2 and Re), were hardly inhibit the hCES1A-mediated DME hydrolysis and hCES2A-mediated FD hydrolysis in HLM even at high dosage (100 μM). By contrast, the ginsenosides without *O*-glycosyl groups (such as Dammarenediol II (DM), panaxadiol, panaxatriol, PPD and PPT), exhibited strong or moderate inhibition on both hCES1A and hCES2A. Of note, 20S-*O*-β-(d-glucosyl)-dammarenediol II (DMG), a ginsenoside with a *O*-glycosyl group at the Rʹ site, also displayed strong inhibition on both hCES1A and hCES2A.

Next, the dose-dependent inhibition curves and the IC_50_ values of each ginsenoside against hCES1A and hCES2A were depicted using varying inhibitor concentrations (Table 1). As shown in Additional file 1: Fig. S1, DM, DMG, panaxadiol and panaxatriol exhibited potent inhibition on the catalytic activities of hCES1A in a dose-dependent manner. The IC_50_ values of DM, DMG, panaxadiol and panaxatriol against hCES1A were 1.99 μM, 1.76 μM, 6.95 μM and 34.93 μM, respectively. Meanwhile, the IC_50_ value of oleanic acid (a known hCES1A inhibitor) against hCES1A in HLM was also determined as 0.10 μM (Additional file 1: Fig. S2). DM, DMG, panaxadiol and panaxatriol also showed strong inhibition on hCES2A, with the IC_50_ values of 0.69 μM, 1.06 μM, 3.78 μM, and 14.59 μM. Meanwhile, the IC_50_ value of LPA (a known hCES2A inhibitor) against hCES2A in HLM was determined as 1.46 μM (Additional file 1: Fig. S2). Compared with LPA, DM, DMG, PPD and PPT showed a stronger inhibition on hCES2A, while PPD and PPT showed high specificity (> 100-folds) toward hCES2A over hCES1A. Furthermore, a number of ginsenosides (e.g., C-K, PPD, PPT, ginsenosides Rh2, F2, Rc, Rg3, F1, Rd, Rh3, Rh4 and F4) showed moderate inhibition on hCES2A, with the IC_50_ values were 15.46 μM, 1.34 μM, 0.86 μM, 5.35 μM, 14.37 μM, 81.66 μM, 10.37 μM, 37.08 μM, 14.05 μM, 5.85 μM, 62.85 μM and 45.02 μM, respectively (Additional file 1: Fig. S3). Meanwhile, the inhibition potential of PPT (a highly specific and potent ginsenoside-type hCES2A inhibitor) on intracellular hCES2A was also investigated in living HepG2 cells. The results clearly showed that PPT was cell-permeable and could inhibit intracellular hCES2A in living cells, with the IC_50_ value of 4.24 μM (Additional file 1: Fig. S4). These results suggested that some ginsenosides showed moderate to strong inhibition on hCES1A and hCES2A, which prompted us to further research the inhibitory mechanism of these ginsenosides on hCES.

**Table 1:** The inhibitory effects of ginsenosides on human carboxylesterases in HLM

No.	Compounds	MW	IC_50_ (μM)		Specificity (hCES1A/hCES2A)
			hCES1A	hCES2A	
1	DM	444.40	1.99 ± 0.21	0.69 ± 0.08	3.06
2	C-K	622.44	> 100	15.46 ± 1.56	> 6.47
3	Rh2	622.44	> 100	5.35 ± 0.56	> 18.86
4	DMG	606.45	1.76 ± 0.28	1.06 ± 0.21	0.33
5	R1	932.53	> 100	> 100	–
6	F2	478.03	> 100	14.37 ± 1.59	7.26
7	Panaxadiol	460.39	6.95 ± 1.29	3.78 ± 0.39	1.84
8	PPD	460.39	> 100	1.34 ± 0.11	> 75.18
9	Panaxatriol	476.39	34.93 ± 6.16	14.59 ± 1.09	2.39
10	PPT	476.39	> 100	0.86 ± 0.09	> 116.28
11	Rb1	1108.60	> 100	> 100	–
12	Rg2	784.50	> 100	> 100	–
13	Rc	1078.59	> 100	81.66 ± 18.56	>1.22
14	Rb2	1078.59	> 100	> 100	–
15	Re	946.55	> 100	> 100	–
16	Rh1	638.88	> 100	> 100	–
17	Rg3	784.50	> 100	10.37 ± 1.17	> 9.64
18	Rb3	1078.59	> 100	> 100	–
19	F1	638.44	> 100	37.08 ± 5.09	> 2.70
20	Rg1	800.49	> 100	> 100	–
21	Rd	946.55	> 100	14.05 ± 1.78	> 7.12
22	Ro	956.50	> 100	> 100	–
23	F4	766.49	> 100	45.02 ± 6.48	> 2.22
24	Rh3	604.43	> 100	5.85 ± 1.17	> 17.09
25	Rh4	620.43	> 100	62.85 ± 6.72	> 1.49

### Structure–inhibition relationships of ginsenosides as hCES inhibitors

After comprehensively analyzing the inhibitory effects of all tested ginsenosides on both hCES1A and hCES2A, the structure–inhibition relationships could be summarized as follows,

1. For the ginsenosides with skeleton A or skeleton C, introduction of any *O*-glycosyl group(s) on the skeleton (including the R1, R2 and Rʹ sites) will lead to the loss of hCES1A inhibition, with the exception of DMG (IC_50_ = 1.76 μM).
2. For the ginsenosides with skeleton A, replacing the hydroxyl groupby a *O*-glycosyl group or introduction of a *O*-glycosyl group at the R_2_ site will abolish the hCES2A inhibition, such as ginsenoside C-K (IC_50_ = 15.46 μM) *VS* ginsenoside Re (IC_50_ > 100 μM), ginsenoside Rg1 (IC_50_ > 100 μM) and ginsenoside R1 (IC_50_ > 100 μM), PPD (IC_50_ = 1.34 μM) *VS* ginsenoside Rg2 (IC_50_ > 100 μM) and ginsenoside Rh1 (IC_50_ > 100 μM).
3. Addition of a hydroxyl group at the R_3_ site of the ginsenosides with skeleton A significantly reduces the inhibitory effects on hCES1A, such as DM (IC_50_ = 1.99 μM) *VS* PPD (IC_50_ > 100 μM), and DMG (IC_50_ = 1.76 μM) *VS* ginsenoside C-K (IC_50_ > 100 μM).
4. For the ginsenosides with skeleton B, introduction of a hydroxyl group at the R_2_ site reduces inhibitory effects on both hCES1A and hCES2A (about 3.8–5.0-folds), such as panaxadiol (IC_50_ = 6.95 μM) *VS* panaxatriol (IC_50_ = 34.93 μM) for hCES1A, and panaxadiol (IC_50_ = 3.78 μM) *VS* panaxatriol (IC_50_ = 14.59 μM) for hCES2A.
5. For the ginsenosides with skeleton A, replacing the hydroxyl group at the Rʹ site by a *O*-glycosyl group does not affect hCES1A inhibition but reduces hCES2A inhibition, such as DM (IC_50_ = 0.69 μM) *VS* DMG (IC_50_ = 1.06 μM), PPT (IC_50_ = 0.86 μM) *VS* ginsenoside F1 (IC_50_ = 37.08 μM), Rh2 (IC_50_ = 5.35 μM) *VS* F2 (IC_50_ = 14.37 μM), as well as PPD (IC_50_ = 1.34 μM) *VS* C-K (IC_50_ = 15.46 μM).
6. For the ginsenosides with skeleton A and skeleton B, the hydroxyl group at the R_1_ site is essential for hCES2A inhibition, the presence of a monosaccharide or polysaccharide group at this site will decrease hCES2A inhibition activity, such as PPD (IC_50_ = 1.34 μM) *VS* Rg3 (IC_50_ = 10.37 μM), PPD (IC_50_ = 1.34 μM) *VS* Rh2 (IC_50_ = 5.35 μM).
7. For the ginsenosides with skeleton A, introduction of a hydroxyl group at the R_3_ site will decrease hCES2A inhibition, such as DM (IC_50_ = 0.69 μM) *VS* PPD (IC_50_ = 1.34 μM), and DMG (IC_50_ = 1.06 μM) *VS* C-K (IC_50_ = 15.46 μM).

Collectively, introduction of the *O*-glycosyl group(s) on the ginsenoside basic skeleton (including the R_1,_ R_2_ and R_3_ sites) is unbeneficial for hCES1A inhibition, while introduction of the *O*-glycosyl group(s) at the R_2_ site on skeleton A will lead to the loss of hCES2A inhibition. Replacing the *O*-glycosyl group at the Rʹ site by a hydroxyl group will enhance the inhibition potency of hCES2A and the specificity over hCES1A, but introduction of a hydroxyl group at the R_3_ site will decrease hCES2A inhibition. These findings will be of advantage to the design and development of more potent and specific ginsenoside-type inhibitors against hCES2A.

### Inhibition kinetics of ginsenosides on hCES

**Fig. 3:**
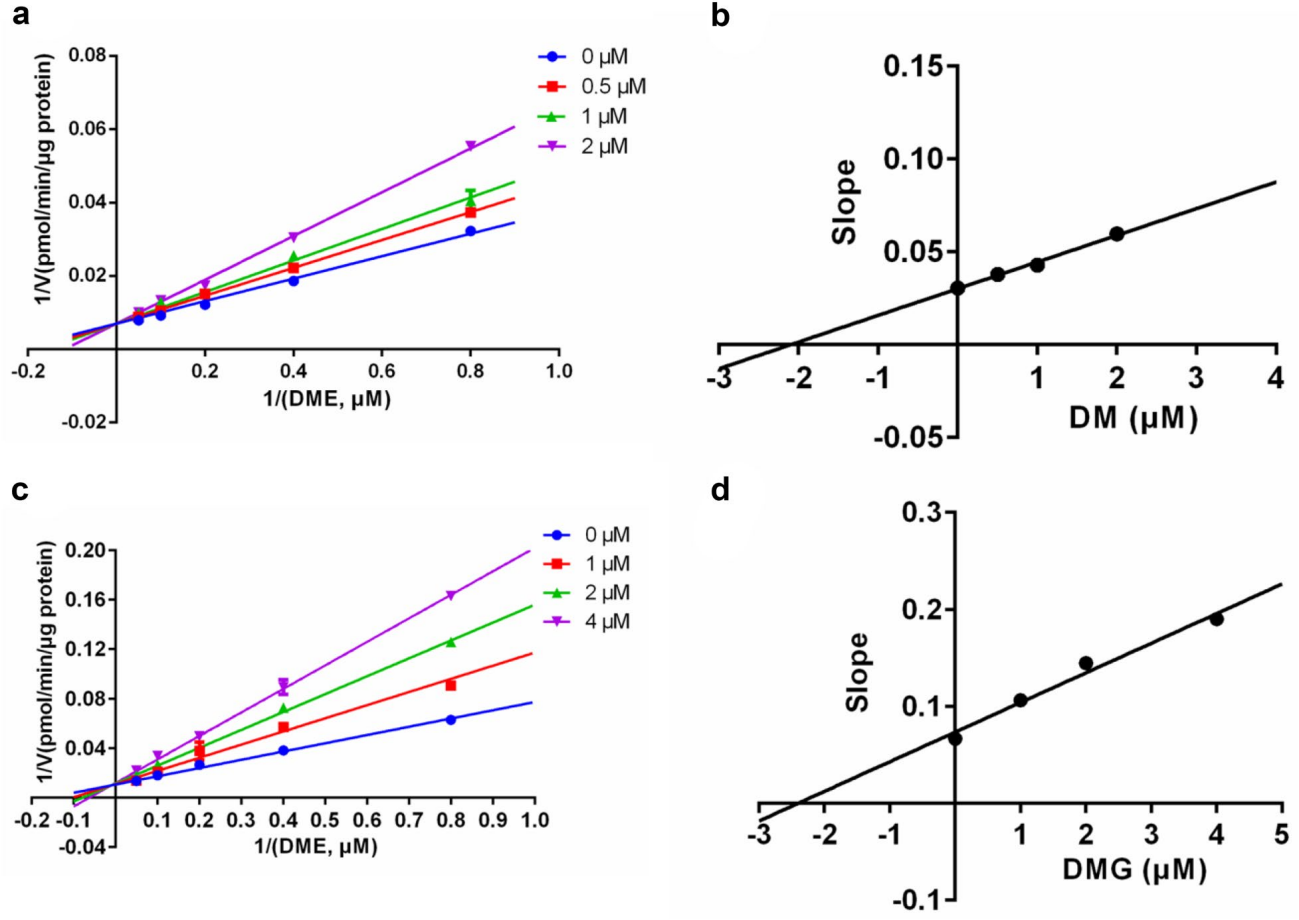
The inhibition kinetic plots of DM and DMG against hCES1A-mediated DME hydrolysis in recombinant HLM. **a** Lineweaver–Burk plots of DM; **b** the corresponding second plot of **a**; **c** Lineweaver–Burk plots of DMG; **d** the corresponding second plot of **c**

**Fig. 4:**
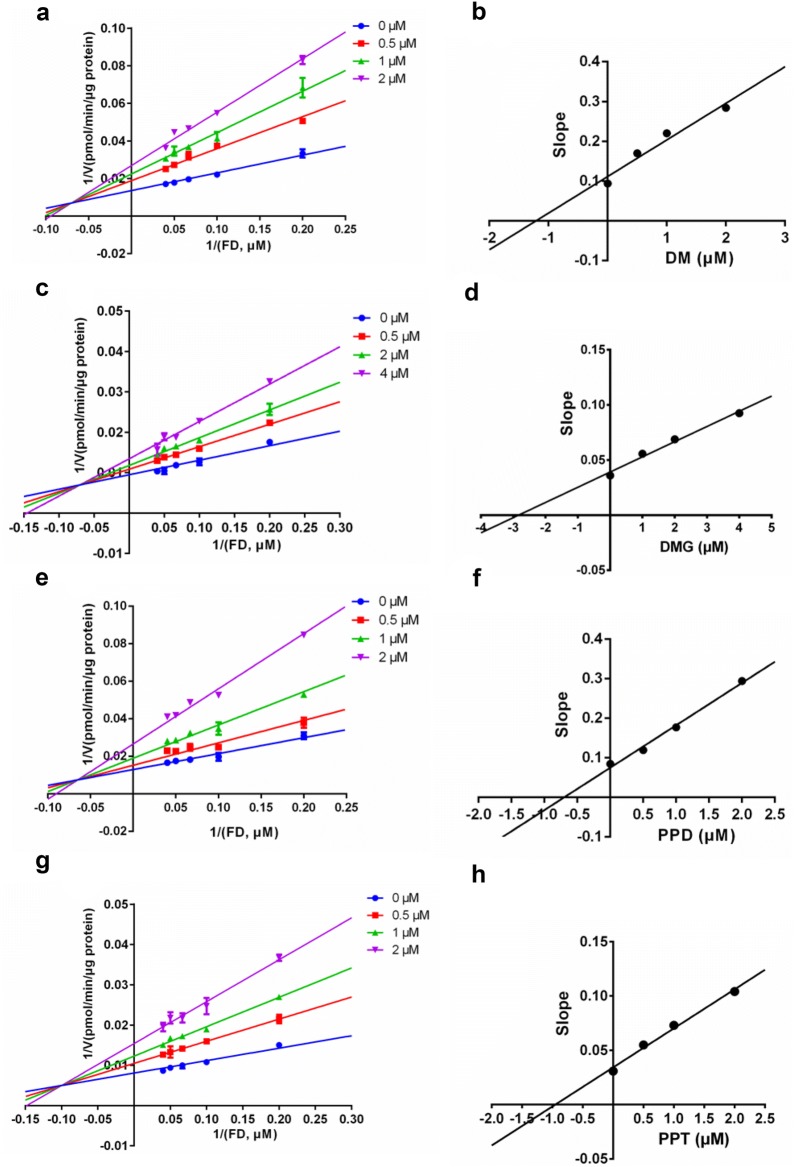
The inhibition kinetic plots of DM, DMG, PPD and PPT against hCES2A-mediated FD hydrolysis in recombinant HLM. **a** Lineweaver–Burk plots of DM; **b** the corresponding second plot of **a**; **c** Lineweaver–Burk plots of DMG; **d** the corresponding second plot of **c**; **e** Lineweaver–Burk plots of PPD; **f** the corresponding second plot of **e**; **g** Lineweaver–Burk plots of PPT; **h** the corresponding second plot of **g**

In this study, several strong ginsenoside-type hCES inhibitors was used to further investigate the inhibition kinetic behaviors of these compounds against hCES, which would facilitate to deeply understand the interactions between ginsenosides and hCES. As shown in Fig. 3, hCES1A-catalyzed DME hydrolysis in HLM could be inhibited by both DM and DMG via competitive inhibition manner, with the *K*_*i*_ values of 2.10 μM and 2.40 μM, respectively (Table 2). This finding indicated that DM and DMG might bind on the identical ligand-binding site in the catalytic cavity of hCES1A, and the binding area of DM and DMG was highly overlapped with that of the substrate DME. Similarly, the inhibition constants and the inhibition modes of four potent ginsenoside-type hCES2A inhibitors were also investigated using FD hydrolysis as probe reaction. As shown in Fig. 4, the Lineweaver–Burk plots clearly showed that DM, DMG, PPD, and PPT inhibited hCES2A-catalyzed FD hydrolysis via a mixed-inhibition manner, with the *K*_*i*_ values of 1.22 μM, 2.83 μM, 0.70 μM and 0.95 μM (Table 2), respectively. These findings suggested that DM, DMG, PPD, and PPT could bind on hCES2A at two distinct ligand-binding sites, which was much different from the binding modes of DM and DMG on hCES1A. Additionally, these findings also suggested that PPD and PPT are particularly strong and highly selective inhibitors of hCES2A, with the *K*_*i*_ values of less than 1.0 μM.

**Table 2:** The inhibition parameters and inhibition modes of potent ginsenoside-type hCES inhibitors

Enzyme source	Target enzyme	Inhibitor	IC_50_ (μM)	*K*_*i*_ (μM)	Inhibition mode	Goodness of fit (R^2^)
HLM	hCES1A	DM	1.99 ± 0.21	2.10	Competitive	0.99
		DMG	1.76 ± 0.28	2.40	Competitive	0.98
HLM	hCES2A	DM	0.69 ± 0.08	1.22	Mixed	0.96
		DMG	1.06 ± 0.21	2.83	Mixed	0.99
		PPD	1.34 ± 0.11	0.70	Mixed	0.99
		PPT	0.86 ± 0.09	0.95	Mixed	0.99

### Docking simulations

**Fig. 5:**
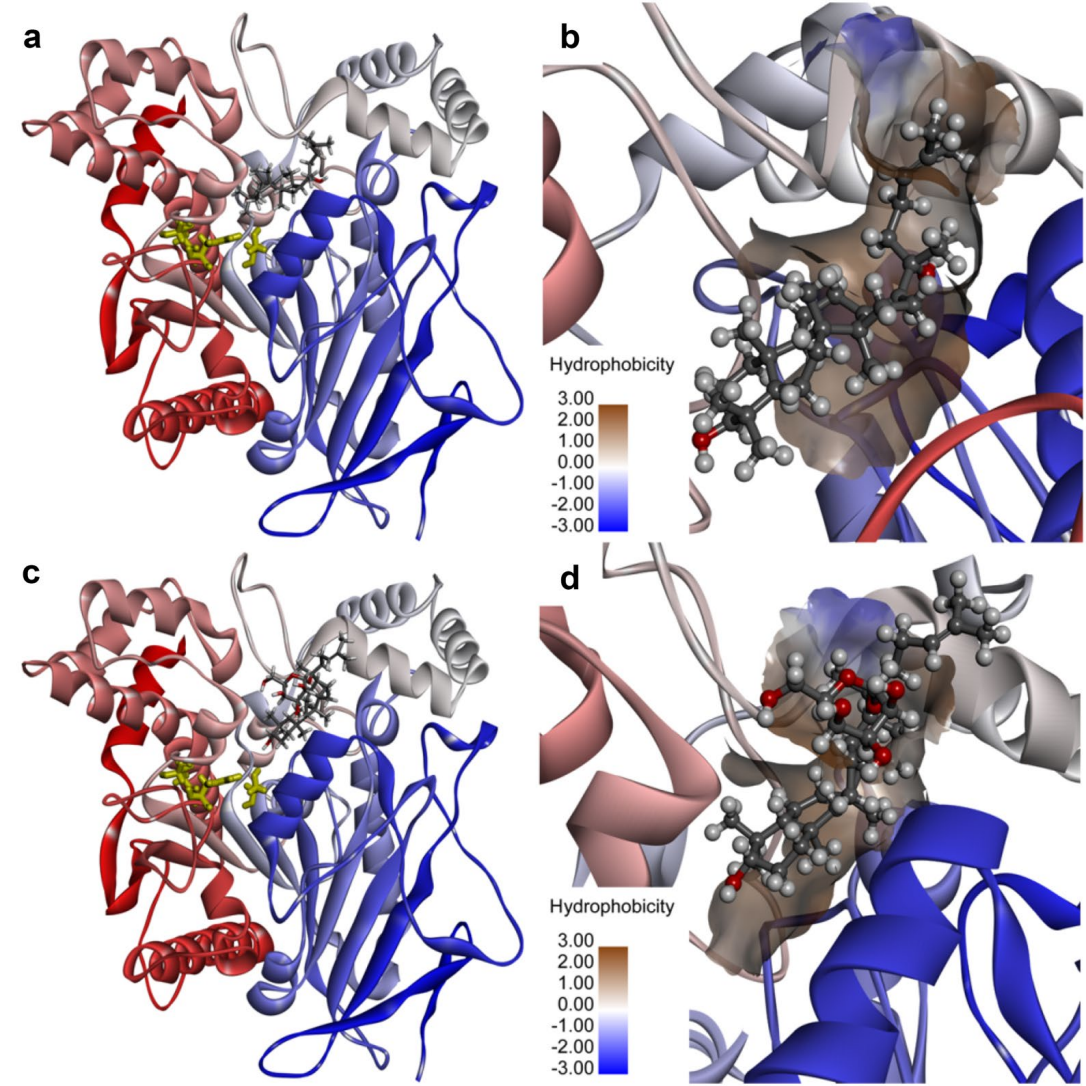
Docking simulations of hCES1A (PDB ID: 1MX5) in the active site. **a** The stereo diagram of DM, **b** a detailed view of the binding area of DM. **c** The stereo diagram of DMG, **d** a detailed view of the binding area of DMG. Note that the catalytic triad of hCES1A (SER^221^, GLU^354^ and HIS^468^) are shown as yellow sticks, the surface hydrophobicity scale in left panel is given from brown (3.0) to blue (− 3.0)

To deeply understand the mechanisms of action of these ginsenosides against both hCES1A and hCES2A, molecular docking simulations of several representative ginsenoside-type hCES inhibitors into hCES were performed by using a previously reported crystal structure of hCES1A (PDB ID: 1MX5) and a modeling hCES2A structure (accession number O00748) as basic models [46, 47]. As shown in Fig. 5 and Additional file 1: Fig. S5, both DM and DMG could be well-docked into the catalytic cavity as well, and their binding areas on hCES1A were highly overlapped with that of the substrate DME. As shown in Additional file 1: Fig. S6, DM could create strong interactions with a panel of residuals in the active site of hCES1A via Van der Waals interactions, alkyl interactions and π-alkyl interactions. Similarly, DMG could strongly interact with ASP^90^ via carbon hydrogen bonding, as well as with LEU^304^, ALA^93^, VAL^146^, MET^145^, PHE^303^, LYS^302^ and LEU^299^ via alkyl interactions or π-alkyl interactions. In addition, it was also found that the presence of *O*-glycosyl group at the Rʹ site was beneficial for the binding of DMG on hCES1A, owing to this group could interact with LYS^92^ and LEU^363^ via conventional hydrogen bonding. These findings showed that DM, DMG and DME could bind at the catalytic site of hCES1A, which is in agreement with the experimental data from inhibition kinetic analyses in which DM and DMG were strong and competitive inhibitors against hCES1A-mediated DME hydrolysis.

**Fig. 6:**
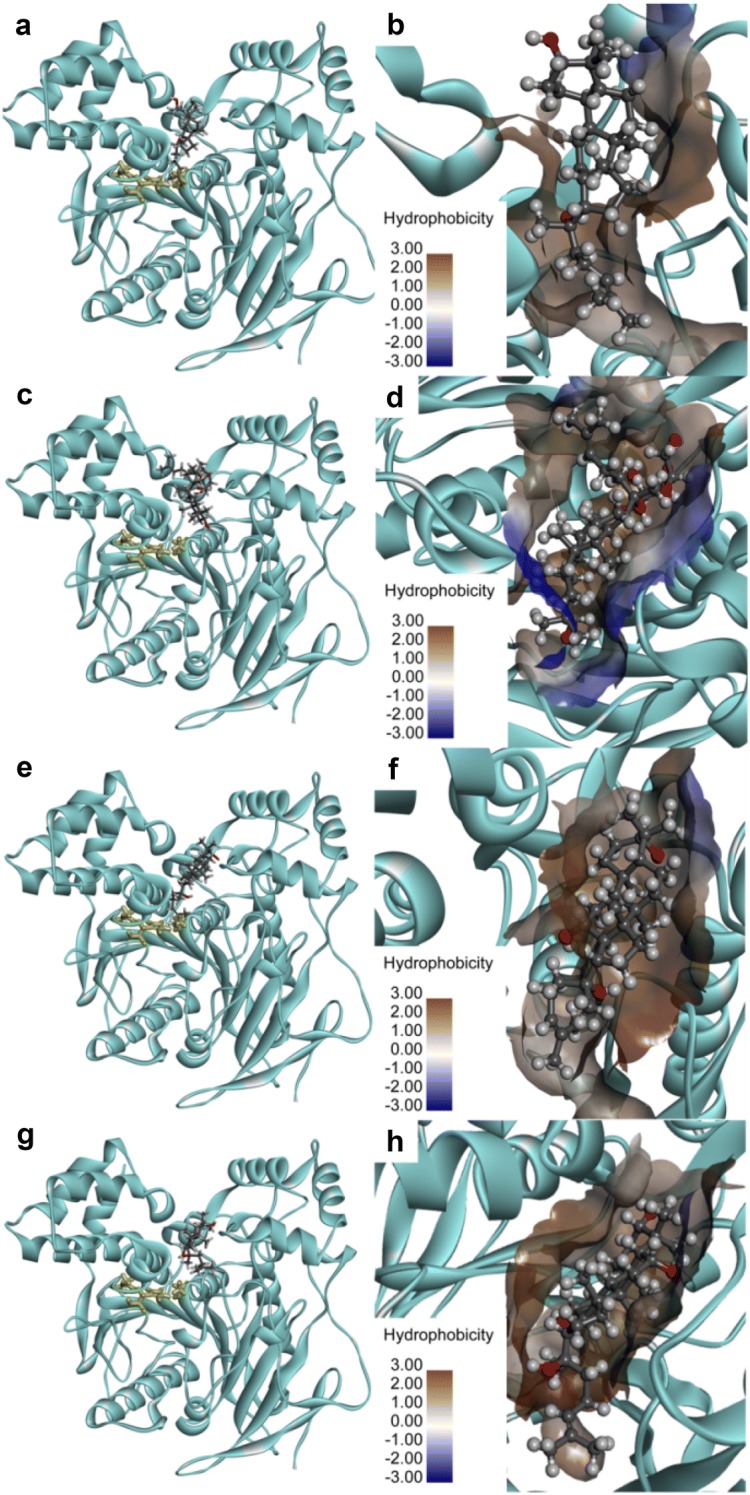
Docking simulations of DM, DMG, PPD, PPT in the active site of hCES2A (UniProt O00748, build by Swiss-model homology model). The stereo diagram of DM (**a**), DMG (**c**), PPD (**e**), PPT (**g**). A detailed view of the binding area of DM (**b**), DMG (**d**), PPD (**f**), PPT (**h**) with surrounding residues. Note that the catalytic triad of hCES2A (SER^228^, GLU^345^ and HIS^457^) are shown as yellow sticks, the surface hydrophobicity scale in left panel is given from brown (3.0) to blue (− 3.0)

**Fig. 7:**
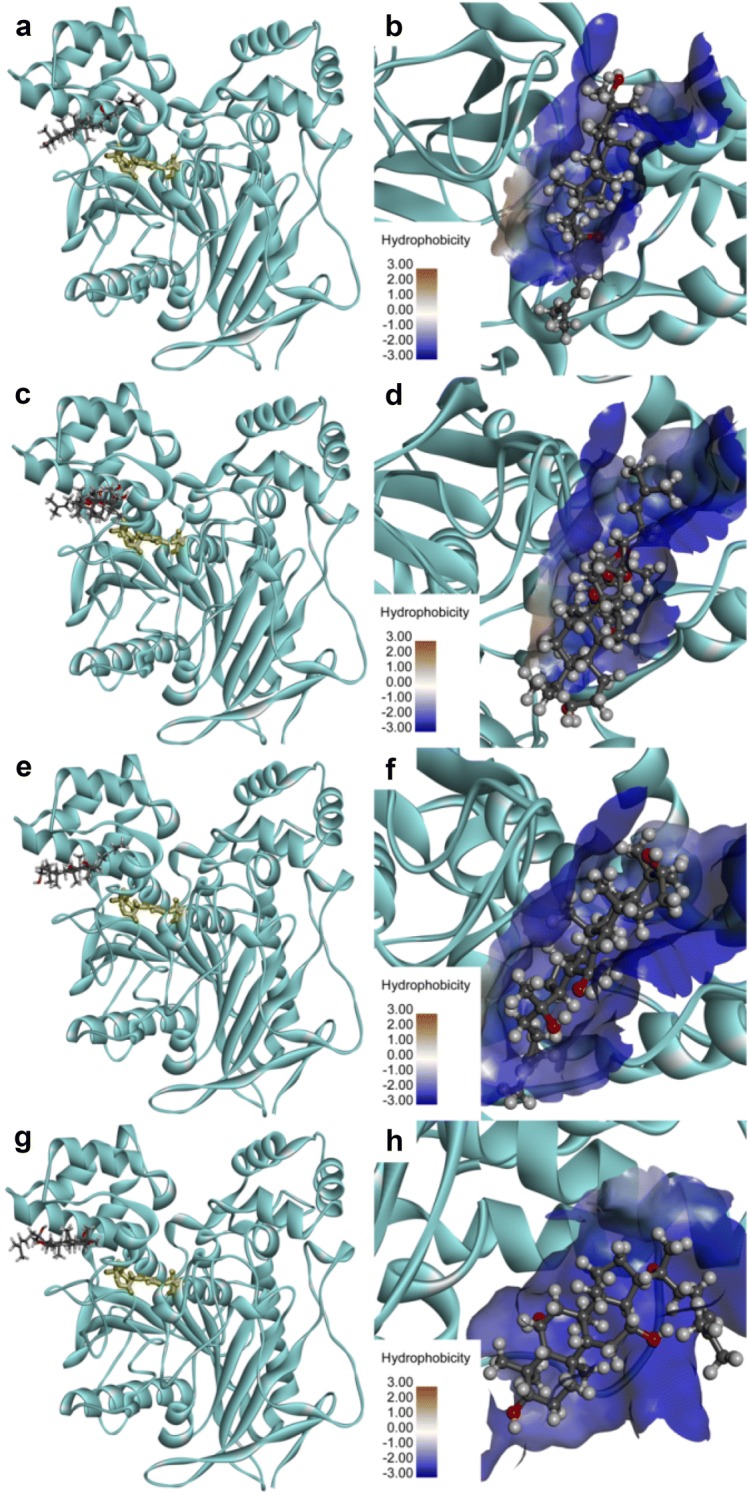
Docking simulations of DM, DMG, PPD, PPT in the Z site of hCES2A (UniProt O00748, build by Swiss-model homology model). The stereo diagram of DM (**a**), DMG (**c**), PPD (**e**), PPT (**g**). A detailed view of the binding area of DM (**b**), DMG (**d**), PPD (**f**), PPT (**h**) with surrounding residues. Note that the catalytic triad of hCES2A (SER^228^, GLU^345^ and HIS^457^) are shown as yellow sticks, the surface hydrophobicity scale in left panel is given from brown (3.0) to blue (− 3.0)

As shown in Figs. 6, 7, molecular docking simulations demonstrated that DM, DMG, PPD and PPT could bind on two distinct ligand-binding sites of hCES2A, one was located in the catalytic cavity that was highly overlapped with that of FD on hCES2A (Additional file 1: Fig. S7), and the other was on the regulatory domain that was far away from the catalytic cavity of hCES2A. These findings agreed well with the mixed-inhibition modes of these four ginsenosides against hCES2A, due to these ginsenosides could act as both competitive inhibitors and non-competitive inhibitors of hCES2A via binding on two distinct ligand-binding sites. The key interactions between these four ginsenosides and hCES2A (including the catalytic cavity and the regulatory domain) were also analyzed. As shown in Additional file 1: Figs. S8–S13, DM, DMG, PPD and PPT could interact with hCES2A at the catalytic cavity or the regulatory domain (Z site) to form relatively stable and low-energy conformations, via a variety of forces including conventional hydrogen bonding, carbon hydrogen bonding, alkyl interactions and π-alkyl interactions. Notably, it seemed that the hydroxyl groups at C-2 site and C-7 site strongly affected the specificity of PPD or PPT on hCES2A. The hydroxyl group at C-2 site of PPD could create strong interactions with MET^309^ via hydrogen bonding in the active site, while the hydroxyl groups at C-2 site and C-7 site of PPT could create strong interactions with several key residuals located in the active site or the Z site of hCES2A, such as MET^309^, PRO^260^ or GLN^408^, via hydrogen bonding. But the similar effects were not shown between hCES1A and hydroxyl groups at C-2 site and C-7 site. As a result, the LibDockScores of these four ginsenosides on hCES2A binding on two distinct ligand-binding sites are similar to each other (Additional file 1: Table S1), which was consistent with the inhibition potency of DM, DMG, PPD and PPT against hCES2A-mediated FD hydrolysis.

## Discussion

Over the past decade, the biological functions of mammalian CES and their links to human diseases have been extensively studied, especially their key roles in the hydrolytic metabolism of both endogenous and xenobiotic esters [48–52]. Accumulating evidence have indicated that hCES1A regulates lipid metabolism and cholesterol homeostasis via hydrolyzing some key endogenous esters including cholesterol esters and triacylglycerols, thereby acts as a key therapeutic target for the treatment of hyperlipidemia, obesity and type 2 diabetes [53, 54]. Furthermore, hCES1A is also a key hydrolase responsible for the hydrolytic metabolism of various ester drugs, such as clopidogrel and oseltamivir [28, 55, 56]. By contrast, hCES2A is a key xenobiotic-metabolizing enzyme in the activation of some frequently used prodrugs, such as irinotecan and capecitabine [57–59]. Notably, hCES2A inhibitor therapy has been considered as one of the feasible strategies for alleviating the intestinal toxicity triggered by irinotecan, which can be activated by hCES2A to the active metabolite SN-38, since hCES2A inhibitors can partially block the over-production of SN-38 in the gastrointestinal system and thereby ameliorate irinotecan associated life-threatening diarrhea [60]. Recent studies have shown that a wide range of natural compounds (such as flavonoids and triterpenoids) isolated from herbs or medicinal plants are strong inhibitors of hCES, and may regulate pharmacokinetic behaviors or lipid metabolism the of hCES-substrate drugs [61–63]. However, the inhibitory effects of ginseng ingredients on hCES have not been well-investigated yet.

As an edible herb, ginseng and its products have been widely used as health-promoting foods or dietary supplements for a long time [64, 65]. Thus, humans can be easily exposed to ginseng ingredients via oral administration of ginseng products. In this study, our findings clearly demonstrated that among all tested ginsenosides, only DM and DMG showed strong inhibition on hCES1A, while other ginsenosides including the microbial metabolites of natural ginsenosides hardly inhibit hCES1A. Thus, it is easily conceivable that ginseng products may hardly trigger herb-drug interactions (HDI) or herb-endobiotic interactions (HEI) via inhibition of hepatic hCES1A. By contrast, a panel of ginsenosides (including Rd and F2, and the microbial metabolites of natural ginsenosides, such as C-K, PPD and PPT) exhibited strong inhibition on hCES2A. Considering that the intestinal hCES2A is a crucial target to ameliorate irinotecan-triggered intestinal toxicity, and the local exposure of ginsenosides in the gastrointestinal system may reach to a high level when the patients repeatedly take ginseng products orally, the ginsenosides may block the over-production of SN-38 in the gastrointestinal system and thereby ameliorate irinotecan-triggered gut toxicity. Of note, a recent study revealed that *Panax notoginseng* saponins could inhibit both hCES1A and hCES2A and down-regulate the protein levels of these two enzymes in Caco-2 cells, therefore affecting hCES-mediated aspirin hydrolysis in Caco-2 cells [66]. This result combined with our findings suggests that the ginseng products may modulate the activity and expression of hCES2A and then affect the pharmacokinetic behaviors of hCES2A substrates, which may be beneficial to patients receiving irinotecan.

From the viewpoint of chemical structure, the ginsenosides have several hydroxyl groups that could be easily modified to generate a series of derivatives. On the basis of SAR of ginsenosides as hCES2A inhibitors, the hydroxyl group at the C-13 site is beneficial for the substrate specificity on hCES2A over hCES1A, which can be used for the development of novel ginsenoside-type hCES2A inhibitors with excellent specificity. Meanwhile, our findings show that the introduction of any *O*-glycosyl group(s) on the skeleton A or skeleton C (including the C-2 and C-7 sites) may lead to the loss of hCES1A inhibition, suggesting that the *O*-glycosyl group at the C-2 and C-7 sites should be replaced by other groups that may create strong interactions with hCES1A. Furthermore, in view of the naturally occurring ginsenosides could be easily available via isolation from herbs or using synthetic biology techniques [67, 68], the medicinal chemists can use the naturally occurring ginsenosides as the starting materials to generate a series of ginsenoside derivatives with good structural connectivity [67, 69], which may strongly facilitate the detailed structure–activity relationship studies and the optimization of both potency and specificity towards hCES2A. Particularly, it is better to generate a wide range of semi-synthetic ginsenoside derivatives with good structural connectivity, via modification of the hydroxyl groups of PPT or PPD at different sites. In the future, some strategies to simultaneously improve the potency and specificity towards hCES2A, such as introducing 3-*O*-β-carboxypropionyl or ethyl ester on these ginsenosides, should be used for the development of next generation hCES2A inhibitors bearing the ginsenoside skeleton [70]. In addition, the computer-aided virtual screening and design strategy could be used to design and develop ginsenoside-type hCES2A inhibitors, on the basis of the structure properties of known hCES inhibitors and the structural features of both hCES1A and hCES2A [37, 71, 72].

## Conclusion

In summary, the inhibitory effects of 25 ginsenosides on hCES as well as the structure–inhibition relationships of ginsenosides as hCES inhibitors, were revealed for the first time. The results clearly demonstrated that the hydroxyl group at the C-13 site is beneficial for the substrate specificity on hCES2A, while the introduction of any *O*-glycosyl group(s) on the skeleton A or skeleton C (including the C-2 and the C-7 sites) would lead to the loss of hCES1A inhibition. Among all tested ginsenosides, DM and DMG displayed relatively strong inhibitions on both hCES1A and hCES2A. Moreover, the gut metabolites of naturally occurring ginsenosides (such as PPD and PPT) exhibited potent and highly specific inhibition on hCES2A, suggesting that these two compounds could serve as ideal lead compounds to develop novel hCES2A inhibitors. Molecular docking simulations demonstrated that PPD and PPT could be well-docked into both the catalytic cavity and the Z site of hCES2A. In short, our results demonstrated that some ginsenosides exhibit strong to moderate inhibition on hCES2A, which hold great promise in modulating the pharmacokinetic profiles and treatment outcomes of hCES2A-substrate drugs, such as irinotecan. Our findings provided novel insights into the interactions between ginsenosides and CES, which were very helpful for the rational use of traditional herb ginseng in clinic, in order to avoid the potential risks of HDI and HEI via inhibition of hCES.

## Supplementary information

**Additional file 1: Table S1.** The Docking simulations of DM, DMG, PPD and PPT on hCES2A. **Fig S1.** The dose-inhibition curves of DM, DMG, panaxadiol and panaxotriol against hCES1A-mediated DME hydrolysis in HLM. **Fig S2.** The dose-inhibition curves of oleanic acid and LPA. **Fig S3.** The dose-inhibition curves of ginsenosides against hCES2A-mediated FD hydrolysis in HLM. **Fig S4.** The inhibitory effect of PPT on hCES2A in living HepG2 cells. **Fig S5.** Docking simulations of DME into hCES1A in the active site. **Fig S6.** 2D representation of the interactions between DM, DMG and the residuals in the active site of hCES1A. **Fig S7.** The docking simulations of FD into hCES2A in the active site. **Fig S8.** 2D representation of the interactions between DM and the residuals in the active site or the Z site of hCES2A. **Fig S9.** 2D representation of the interactions between DMG and the residuals in the active site or the Z site of hCES2A. **Fig S10.** 2D representation of the interactions between PPD and the residuals in the active site or the Z site of hCES2A. **Fig S11.** 2D representation of the interactions between PPT and the residuals in the active site or the Z site of hCES2A. **Fig S12.** 2D representation of the interactions between PPD and the residuals in the active site or the Z site of hCES1A. **Fig S13.** 2D representation of the interactions between PPT and the residuals in the active site or the Z site of hCES1A.

## Data Availability

Most of the data generated of analyzed during the study are included in this article and its additional information.

## Abbreviations

hCES: human carboxylesterases
CES: carboxylesterases
hCES1A: human carboxylesterases 1A
hCES2A: human carboxylesterases 2A
UGT2B7: UDP-glucuronosyltransferase 2B7
CYP3A4: cytochrome P450 3A4
HLM: human liver microsomes
DM: Dammarenediol II
DMG: 20S-*O*-β-(d-glucosyl)-dammarenediol II
PPD: protopanaxadiol
PPT: protopanaxatriol
LDR: Luciferin Detection Reagent
FD: fluorescein diacetate
NCEN: *N*-(2-butyl-1,3-dioxo-2,3-dihydro-1*H*-phenalen-6-yl)-2-chloroacetamide
LPA: loperamide
DME: d-luciferin methyl ester
HDI: herb–drug interaction
HEI: herb-endobiotic interaction
