# Supplementary material for: Inhibition of human carboxylesterases by ginsenosides: structure–activity relationships and inhibitory mechanism
Source: Chin Med. 2019 Dec 16;14:56. doi: 10.1186/s13020-019-0279-0 (PMC6915887; doi:10.1186/s13020-019-0279-0)
Supplement: Supplementary file 1 — Additional file 1: Table S1. The Docking simulations of DM, DMG, PPD and PPT on hCES2A. Fig S1. The dose-inhibition curves of DM, DMG, panaxadiol and panaxotriol against hCES1A-mediated DME hydrolysis in HLM. Fig S2. The dose-inhibition curves of oleanic acid and LPA. Fig S3. The dose-inhibition curves of ginsenosides against hCES2A-mediated FD hydrolysis in HLM. Fig S4. The inhibitory effect of PPT on hCES2A in living HepG2 cells. Fig S5. Docking simulations of DME into hCES1A in the active site. Fig S6. 2D representation of the interactions between DM, DMG and the residuals in the active site of hCES1A. Fig S7. The docking simulations of FD into hCES2A in the active site. Fig S8. 2D representation of the interactions between DM and the residuals in the active site or the Z site of hCES2A. Fig S9. 2D representation of the interactions between DMG and the residuals in the active site or the Z site of hCES2A. Fig S10. 2D representation of the interactions between PPD and the residuals in the active site or the Z site of hCES2A. Fig S11. 2D representation of the interactions between PPT and the residuals in the active site or the Z site of hCES2A. Fig S12. 2D representation of the interactions between PPD and the residuals in the active site or the Z site of hCES1A. Fig S13. 2D representation of the interactions between PPT and the residuals in the active site or the Z site of hCES1A. [file 13020_2019_279_MOESM1_ESM.docx]

**Additional Materials**

*for*

**Inhibition of human carboxylesterases by ginsenosides: Structure-activity relationships and inhibitory mechanism**

Zhao-Hui Sun^1^, Jing Chen^2^, Yun-Qing Song^1^, Tong-Yi Dou^2^, Li-Wei Zou^1^, Da-Cheng Hao^3^, Hai-Bin Liu^4^, Guang-Bo Ge^1*^, Ling Yang^1*^

^1^ Institute of Interdisciplinary Integrative Medicine Research, Shanghai University of Traditional Chinese Medicine, Shanghai, 201203, China.

^2^ School of Life Science and Medicine, Dalian University of Technology, Panjin 124221, China.

^3^ School of Environment and Chemical Engineering, Dalian Jiaotong University, Dalian 116028, China.

^4^ National Engineering Research Center for Gelatin-based Traditional Chinese Medicine, Dong-E-E-Jiao Co. Ltd., Liaocheng, 252201, China.

*Corresponding author.

E-mail address:

geguangbo@dicp.ac.cn (G.-B. Ge); yling@shutcm.edu.cn (L. Yang)

telephone and fax numbers:

021-51323184 (G.-B. Ge); 021-51323182 (L. Yang)

**Contents**

**Table S1. The Docking simulations of DM, DMG, PPD and PPT on hCES2A.**

**Fig S1. The dose-inhibition curves of DM, DMG, panaxadiol and panaxotriol against hCES1A-mediated DME hydrolysis in HLM.**

**Fig S2. The dose-inhibition curves of oleanic acid and LPA.**

**Fig S3. The dose-inhibition curves of ginsenosides against hCES2A-mediated FD hydrolysis in HLM.**

**Fig S4. The inhibitory effect of PPT on hCES2A in living HepG2 cells**

**Fig S5. Docking simulations of DME into hCES1A** **in the active site.**

**Fig S6. 2D representation of the interactions between DM, DMG and the residuals in the active site of hCES1A.**

**Fig S7. The docking simulations of FD into hCES2A in the active site.**

**Fig S8. 2D representation of the interactions between DM and the residuals in the active site or the Z site of hCES2A.**

**Fig S9. 2D representation of the interactions between DMG and the residuals in the active site or the Z site of hCES2A.**

**Fig S10. 2D representation of the interactions between PPD and the residuals in the active site or the Z site of hCES2A.**

**Fig S11. 2D representation of the interactions between PPT and the residuals in the active site or the Z site of hCES2A.**

**Fig S12. 2D representation of the interactions between PPD and the residuals in the active site or the Z site of hCES1A.**

**Fig S13. 2D representation of the interactions between PPT and the residuals in the active site or the Z site of hCES1A.**

**Table S1.** The Docking simulations of DM, DMG, PPD and PPT on hCES2A.

| Inhibitor | Active site | | Z site | |
| --- | --- | --- | --- | --- |
|  | Conformation number | LibDockScore | Conformation number | LibDockScore |
| DMG | 64 | 143.048 | 72 | 120.238 |
| DM | 68 | 110.025 | 64 | 120.702 |
| PPD | 54 | 109.665 | 52 | 118.894 |
| PPT | 64 | 112.571 | 66 | 117.453 |
| FD | 45 | 110.185 | 38 | 101.515 |


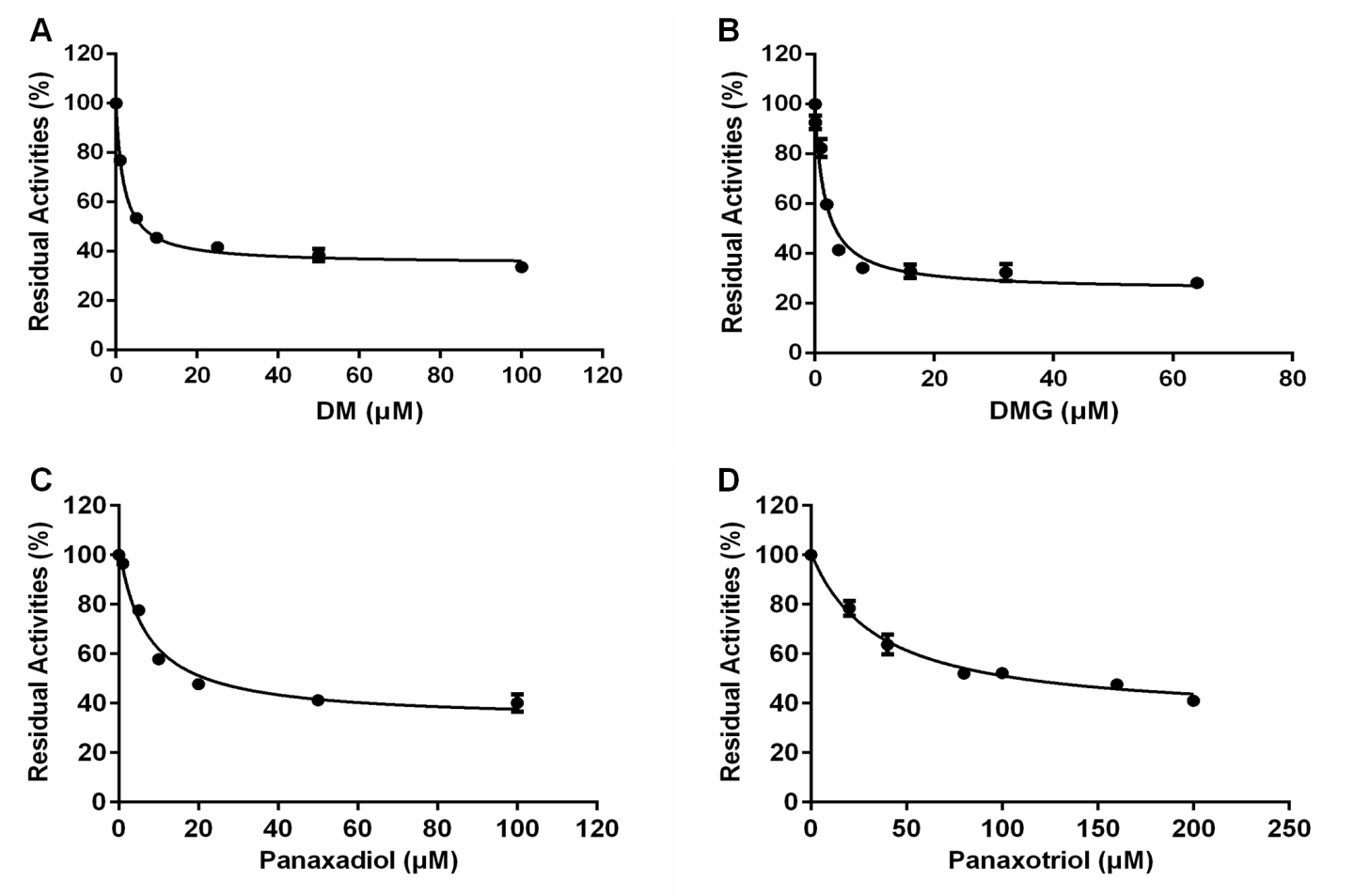


**Fig S1.** The dose-inhibition curves of DM(A), DMG(B), panaxadiol(C) and panaxotriol(D) against hCES1A-mediated DME hydrolysis in HLM.

**Fig S2.** The dose-inhibition curves of oleanic acid and loperamide (LPA). A) oleanic acid was used as a positive control inhibitor of hCES1A with the IC_50_ value of 0.10 ± 0.01 μM, B) LPA was used as a positive control inhibitor of hCES2A with the IC_50_ value of 1.46 ± 0.15 μM.

**Fig S3.** The dose-inhibition curves of ginsenosides against hCES2A-mediated FD hydrolysis in HLM.


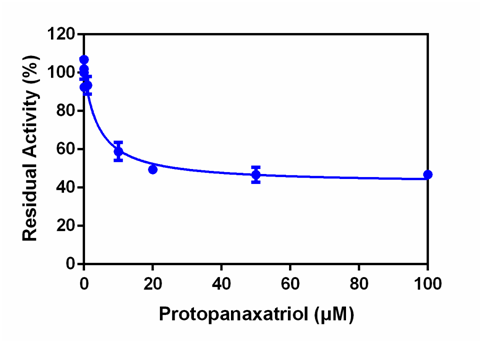


**Fig S4.** The inhibitory effect of PPT on hCES2A in living HepG2 cells with the IC_50_ value of 4.24 ± 1.11 μM.

**
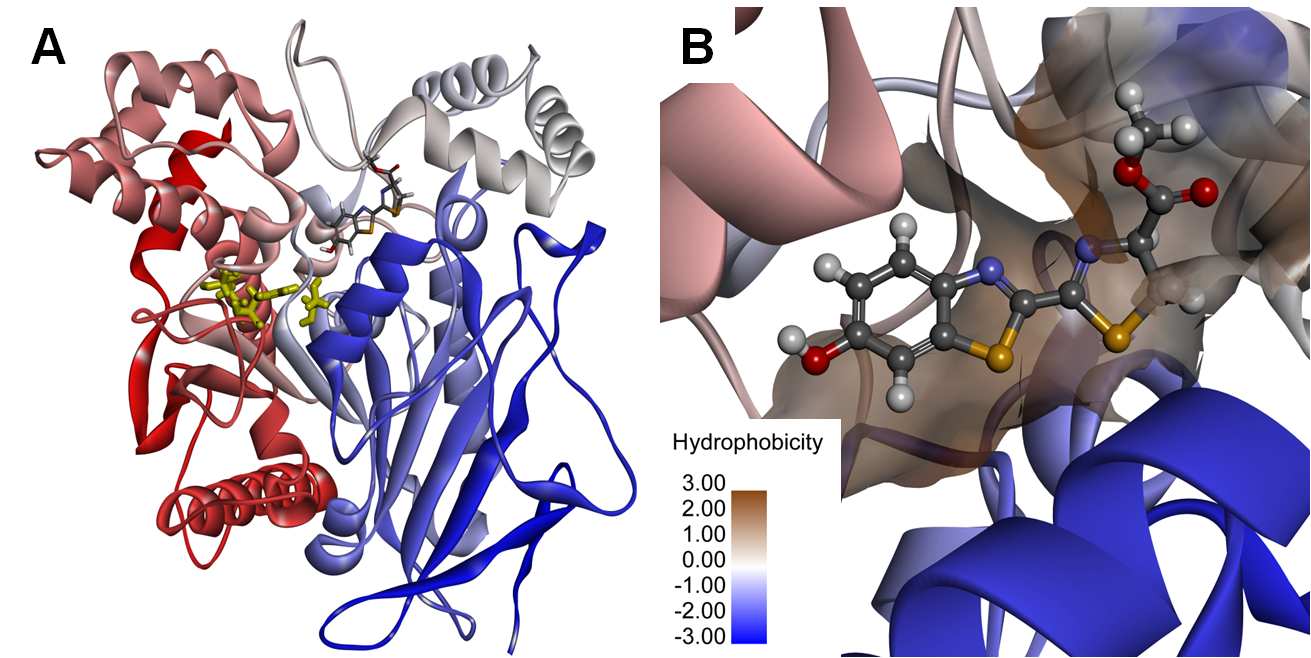
**

**Fig S5**. Docking simulations of DME into hCES1A (PDB ID: 1MX5) in the active site. A) The stereo diagram of DME, B) a detailed view of the binding area of DME. Note that the catalytic triad of hCES1A (SER^221^, GLU^354^ and HIS^468^) are shown as yellow sticks, the surface hydrophobicity scale in left panel is given from brown (3.0) to blue (-3.0).

**
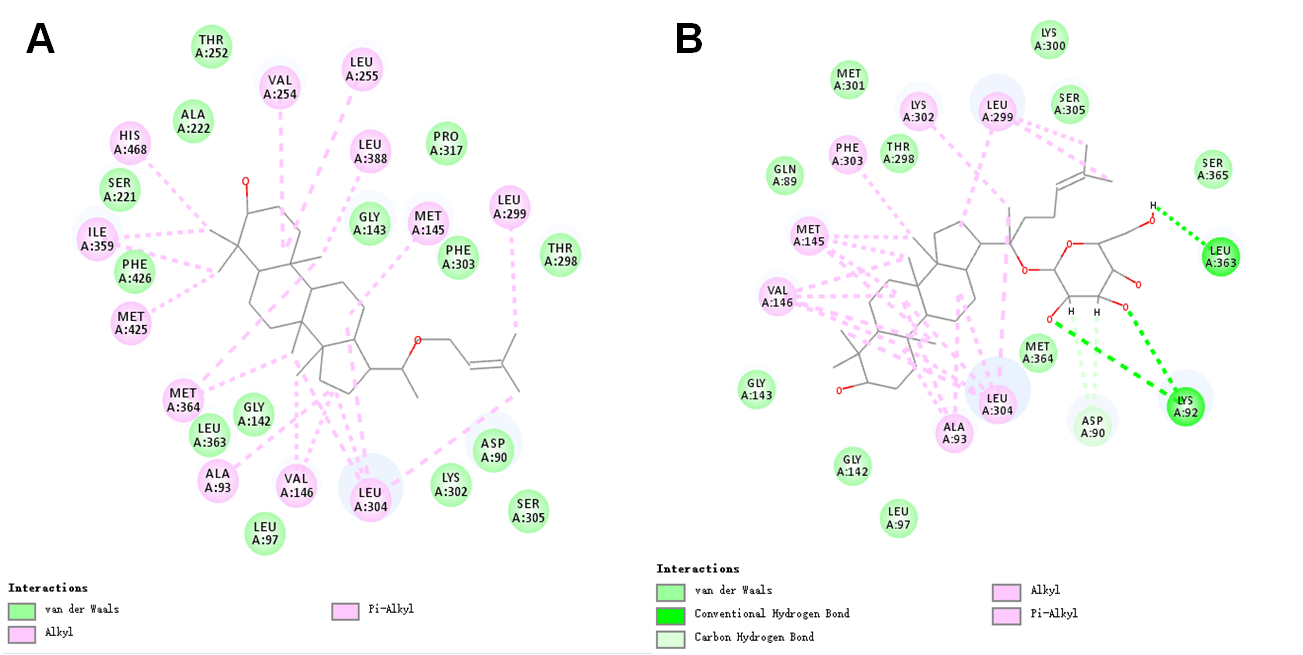
**

**Fig S6.** 2D representation of the interactions between DM, DMG and the residuals in the active site of hCES1A. Note that DM could create strong interactions with a panel of residuals in the active site of hCES1A (A) *via* Van der Waals interactions. By contrast, DMG created strong interactions with ASP^90^ *via* carbon hydrogen bonding, and with LEU^363^ and LYS^92^ in the active site *via* conventional hydrogen bonding (B).

**
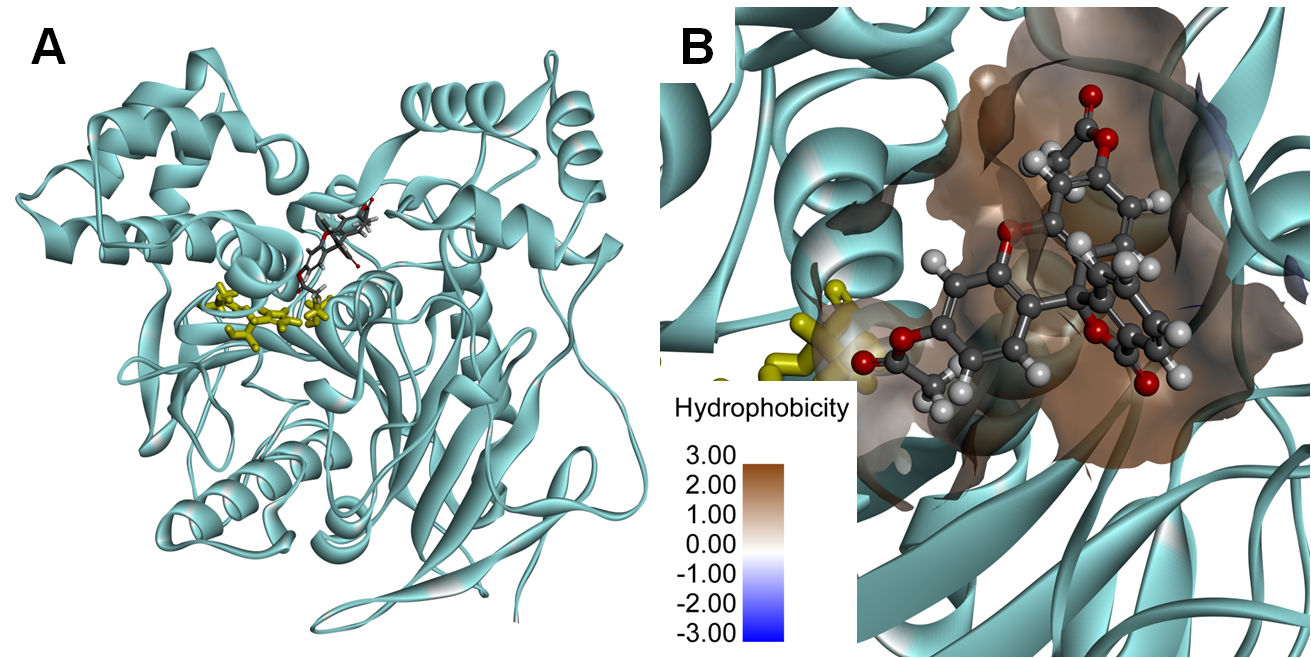
**

**Fig S7.** The docking simulations of FD into hCES2A (UniProt O00748, build by Swiss-model homology model) in the active site. A) The stereo diagram of FD, B) a detailed view of the binding area of FD. Note that the catalytic triad of hCES2A (SER^228^, GLU^345^ and HIS^457^) are shown as yellow sticks, the surface hydrophobicity scale in left panel is given from brown (3.0) to blue (-3.0).


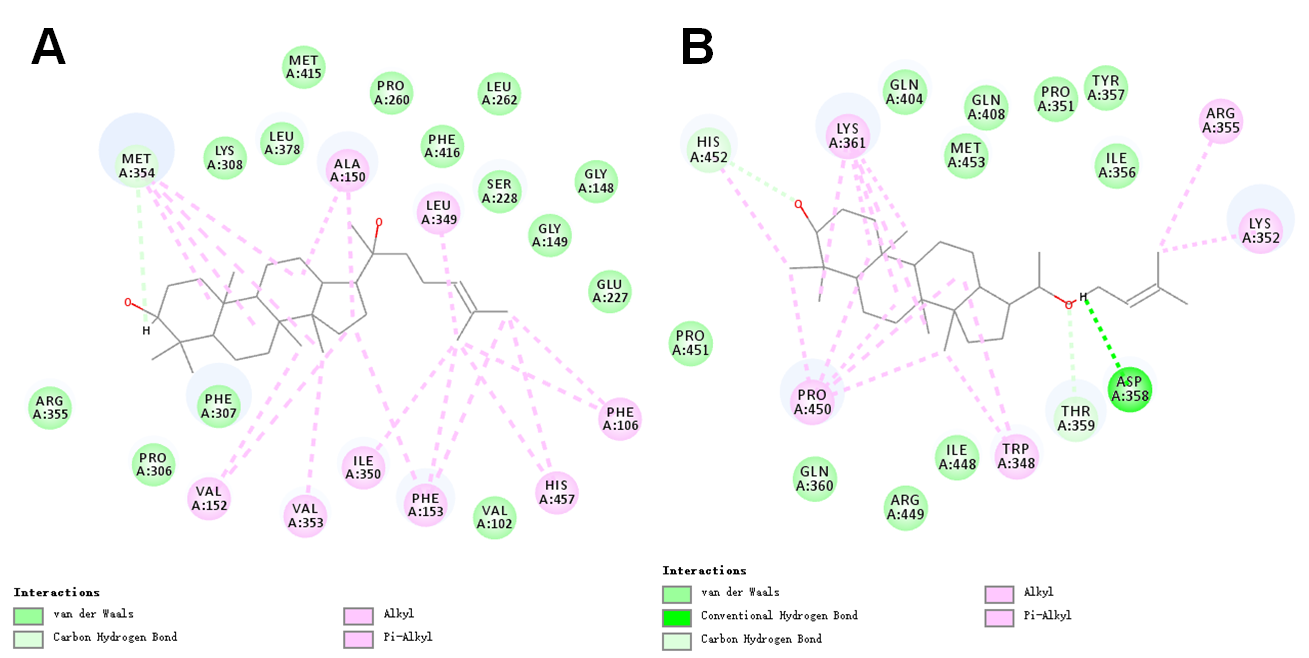


**Fig S8.** 2D representation of the interactions between DM and the residuals in the active site (A) or the Z site (B) of hCES2A. Note that DM created strong interactions with MET^354^ *via* hydrogen bonding and with a panel of residuals in the active site *via* Van der Waals interactions. By contrast, DM could create strong interactions with ASP^358^ ,THR^359^ and HIS^452^ *via* hydrogen bonding in the Z site (B) of hCES2A.


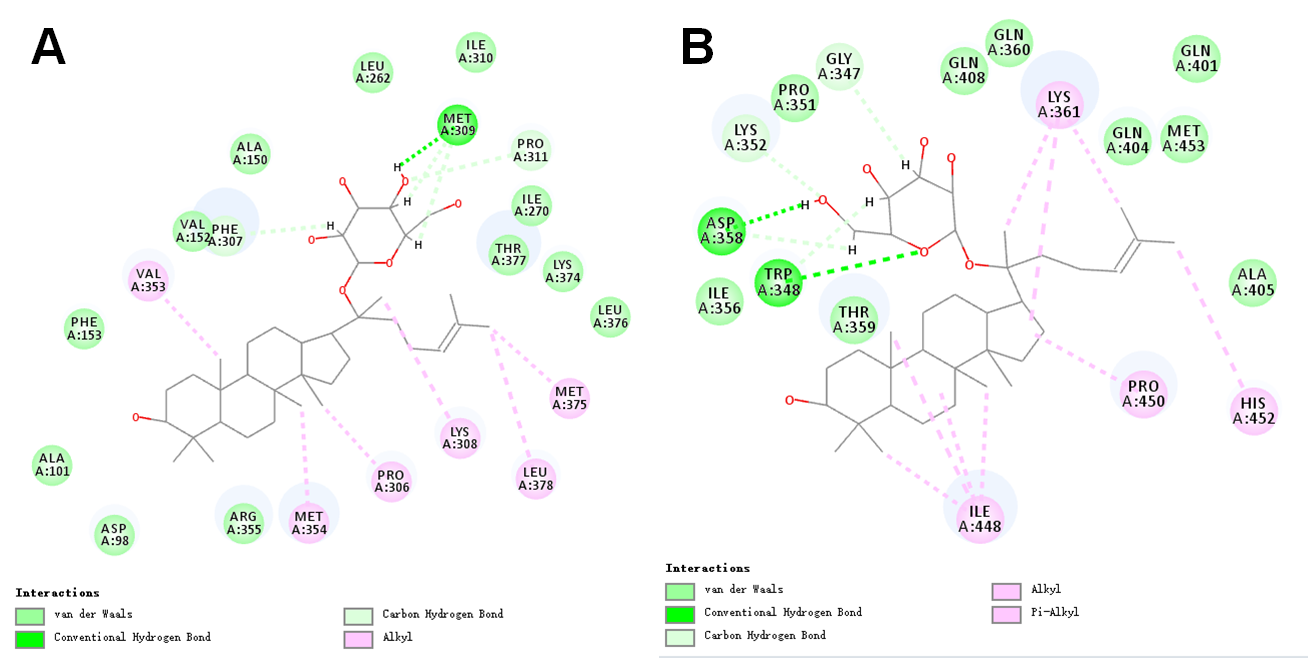


**Fig S9.** 2D representation of the interactions between DMG and the residuals in the active site (A) or the Z site (B) of hCES2A. Note that DMG created strong interactions with PRO^311^, PHE^307^ and MET^309^ *via* hydrogen bonding and with a panel of residuals in the active site *via* Van der Waals interactions. By contrast, DMG could create strong interactions with ASP^358^, TRP^348^, GLY^347^ and LYS^352^ *via* hydrogen bonding in the Z site (B) of hCES2A.


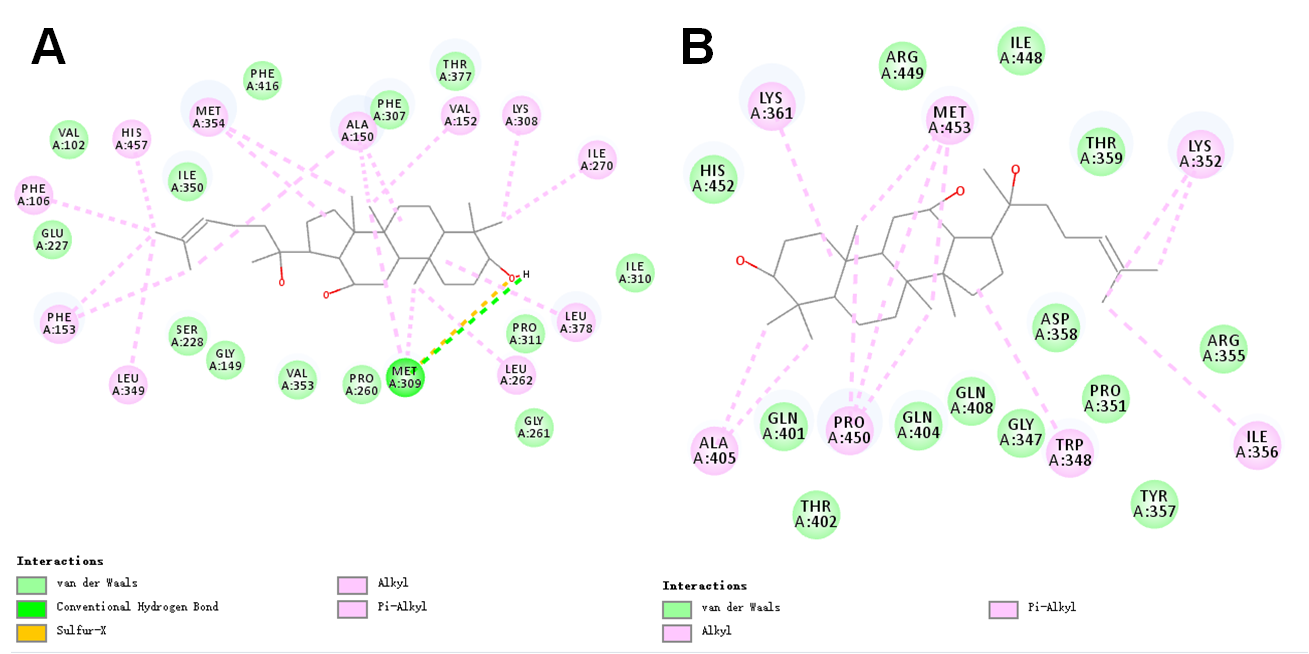


**Fig S10.** 2D representation of the interactions between PPD and the residuals in the active site (A) or the Z site (B) of hCES2A. Note that PPD created strong interactions with MET^309^ *via* hydrogen bonding in the active site. By contrast, PPD could create strong interactions with a panel of residuals *via* Van der Waals interactions in the Z site (B) of hCES2A.


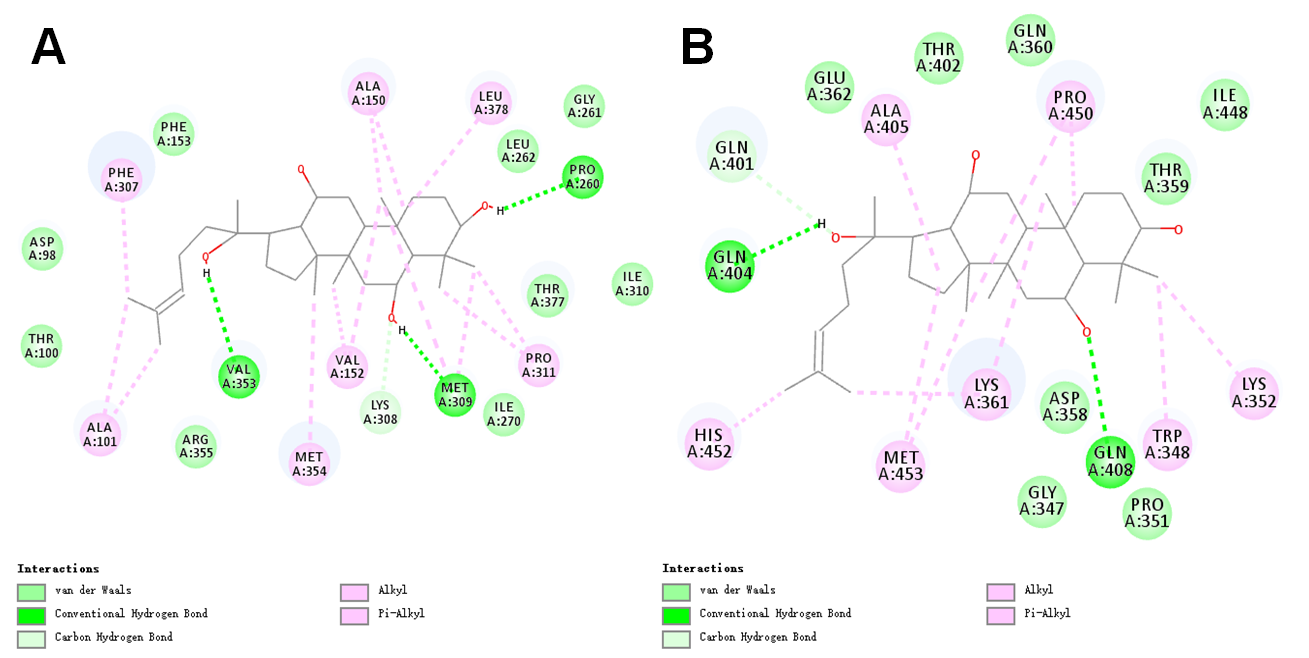


**Fig S11.** 2D representation of the interactions between PPT and the residuals in the active site (A) or the Z site (B) of hCES2A. Note that PPT created strong interactions with MET^309^, PRO^260^, VAL^353^ or LYS^308^ *via* hydrogen bonding and with a panel of residuals in the active site *via* Van der Waals interactions. By contrast, PPT could create strong interactions with GLN^408^, GLN^404^ and GLN^401^ *via* hydrogen bonding in the Z site (B) of hCES2A.

**Fig S12.** 2D representation of the interactions between PPD and the residuals in the active site (A) or the Z site (B) of hCES1A. Note that PPD created strong interactions with a panel of residuals in the active site *via* Van der Waals interactions. By contrast, PPD could create strong interactions with GLY^356^ *via* hydrogen bonding in the Z site (B) of hCES1A.

**Fig S13.** 2D representation of the interactions between PPT and the residuals in the active site (A) or the Z site (B) of hCES1A. Note that PPT created strong interactions with ASP^90^ *via* hydrogen bonding in the active site. By contrast, PPT could create strong interactions with GLY^356^ *via* hydrogen bonding in the Z site (B) of hCES1A.
